# The Positive Effect of ZnS in Waste Tire Carbon as Anode for Lithium-Ion Batteries

**DOI:** 10.3390/ma14092178

**Published:** 2021-04-24

**Authors:** Xuechen Wang, Lu Zhou, Jianjiang Li, Na Han, Xiaohua Li, Gang Liu, Dongchen Jia, Zhaoli Ma, Guojun Song, Xiaoyi Zhu, Zhi Peng, Lei Zhang

**Affiliations:** 1 School of Material Science and Engineering, Qingdao University, No. 308, Ningxia Road, Qingdao 266071, China; 2018020384@qdu.edu.cn (X.W.); 2018020395@qdu.edu.cn (N.H.); 2019020442@qdu.edu.cn (X.L.); songguojunqdu@126.com (G.S.); 2 College of Chemistry and Chemical Engineering, Qingdao University, No. 308, Ningxia Road, Qingdao 266071, China; zhoulu137@126.com; 3 School of Environmental Science and Engineering, Qingdao University, No. 308, Ningxia Road, Qingdao 266071, China; jjli@qdu.edu.cn (J.L.); 2019025785@qdu.edu.cn (G.L.); 2020025847@qdu.edu.cn (D.J.); xyzhu@qdu.edu.cn (X.Z.); 4 Chemical Experimental Teaching Center, Qingdao University, No. 308, Ningxia Road, Qingdao 266071, China; zlma@qdu.edu.cn; 5 Centre for Clean Environment and Energy, Gold Coast Campus, Griffith University, Gold Coast, QLD 4222, Australia

**Keywords:** waste tire carbon, waste tire powder, tire recycling, anode, lithium-ion battery

## Abstract

There is great demand for high-performance, low-cost electrode materials for anodes of lithium-ion batteries (LIBs). Herein, we report the recovery of carbon materials by treating waste tire rubber via a facile one-step carbonization process. Electrochemical studies revealed that the waste tire carbon anode had a higher reversible capacity than that of commercial graphite and shows the positive effect of ZnS in the waste tire carbon. When used as the anode for LIBs, waste tire carbon shows a high specific capacity of 510.6 mAh·g^−1^ at 100 mA·g^−1^ with almost 97% capacity retention after 100 cycles. Even at a high rate of 1 A·g^−1^, the carbon electrode presents an excellent cyclic capability of 255.1 mAh·g^−1^ after 3000 cycles. This high-performance carbon material has many potential applications in LIBs and provide an alternative avenue for the recycling of waste tires.

## 1. Introduction

Globally, approximately 17 million tons of scrap tires are generated per year. It is anticipated that the use of tires will increase to 1200 million annually by 2030 [1,2]. In China alone, the total weight of tires produced between 2011 and 2018 was 172.82 million tons, of which 55.8% were waste tires. Only 37.2% of these waste tires were recovered, thus causing serious environmental issues [3]. If handled incorrectly, it is difficult to avoid unnecessary energy consumption and environmental pollution. When exposed to air, the seepage of chemical compounds from tires gives rise to soil and air pollution [4,5]. Consequently, the development of advanced methods for recovering waste tire rubber is essential to ensure their recycling.

Due to their complicated structure and the presence of various complex additives, waste tires cannot readily decompose and resist degradation under even harsh chemical and physical conditions [6]. Hence, their recycling and reuse is particularly difficult, particularly because either their cross-linked network or the C–C bonds of the main carbon chain certainly are destroyed [7]. Waste tire rubber is a mixture of various elastomers, including natural butadiene, styrene, and butadiene together with additives such as carbon black, sulfur, and ZnO [8]. During simple combustion, waste tires are incompletely treated, and the toxic gases (SO_2_, CO, H_2_S, etc.) generated are harmful to human health and the environment [9,10]. Techniques for effective recycling of waste tires have been extensively developed in order to obtain more environmentally friendly materials. For example, Wei Li et al. obtained derived oil from waste tires by pyrolysis in a continuously stirred batch reactor, both in the presence and absence of catalysts, producing high-value-added chemical materials or fuel oil [11]. Yulin Zhang et al. prepared a hierarchical porous carbon material which exhibits excellent adsorption performance from the heavy residue of waste tire-derived pyrolytic by oil using the template strategy [12]. Yuxin Zhang et al. recovered high-performance rubber composites from reclaimed waste tires using a dynamic thermal-oxidative reclamation reactor [13]. Thus, it is evident that value-added materials can be manufactured from waste tires.

**Scheme 1:**
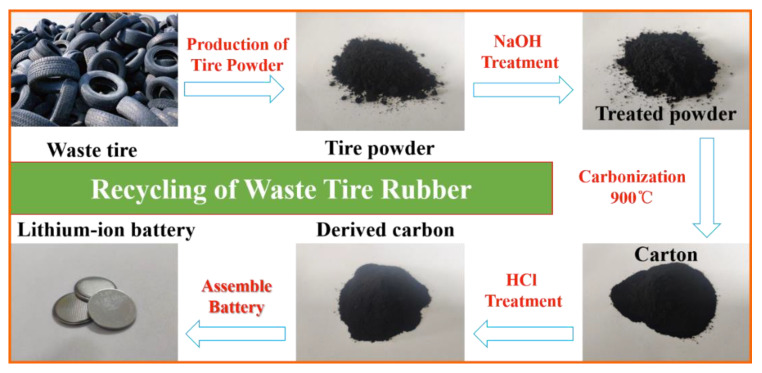
Schematic preparation procedure for waste tire carbon from waste tires.

In this paper, we report a new strategy for producing energy storage materials from waste tires. Scheme 1 demonstrates the preparation of the recovered carbon materials. After intricate cleaning and a simple carbonization process, we extracted carbon material from waste tire powder as anode material of a lithium-ion battery and achieved excellent cycling performance. In the carbonization process, ZnS is naturally wrapped in the waste tire carbon. Compared with common carbon materials, waste tire carbon indicates more stable structure and capacity advantages. This is because: (1) waste tire rubber is designed to be exceptionally stable under harsh conditions by virtue of its crosslinked structure, which is also beneficial to structural stability during cycling [14]. (2) ZnS in situ generated during the high-temperature treatment of waste tire rubber can improve lithium storage performance [15]. For waste tire carbon, we expect it to be used in other electrochemical fields, such as a supercapacitor [16], K-ion battery [17], and other types of batteries [18]. What is the most important is that the material we reported not only alleviates environmental problems to a certain extent, but also develops a kind of carbon material which is easy to obtain and prepare.

## 2. Materials and Methods

### 2.1. Materials

Waste tire rubber powders were obtained from Guangzhou Hongtai Holding Co., Ltd. (Guangzhou, China). and commercial graphite powder was purchased from Qingdao Huatai lubrication and Sealing Technology Co., Ltd. (Qingdao, China). All materials used in the battery test, including acetylene black, polyvinylidene fluoride (PVDF), 1 M LiPF_6_ electrolyte, and battery shells were purchased from the Shenzhen Kejing Zhida Technology Co., Ltd. (Shenzhen, China). Chemicals used during the preparation stage, including hydrochloric acid (AR, HCl), sodium hydroxide (AR, NaOH), and ethanol (AR, 99.7%) were purchased from the Sinopharm Chemical Reagent Co., Ltd. (Shanghai, China). Zinc sulfide (AR, ZnS) was purchased form the Shanghai Macklin Biochemical Co., Ltd. (Shanghai, China).

### 2.2. Preparation

In a typical treatment process, waste tire powders were initially washed with hot sodium hydroxide hydroalcoholic solution to remove surface contamination and the inorganic additive white carbon black. Then, the powders were pyrolyzed under argon at 900 °C for 1 h to obtain a carbon composite, denoted as waste tire carbon-1 (WTC-1). WTC-1 was further washed by using hydrochloric acid hydroalcoholic solution and double-distilled water to remove partial impurities, and the resulting product was termed waste tire carbon-2 (WTC-2). The commercial graphite was selected to compare its electrochemical performance with other composites. Furthermore, in order to verify the role of ZnS, a certain mass ratio (5%) of ZnS and commercial graphite (called ZnS/C) was mixed uniformly by grinding.

### 2.3. Characterization

X-ray diffraction (XRD) patterns of the prepared samples were obtained using a DX-2007 (XRD) apparatus (λ = 1.5418 Å) (Dandong Haoyuan Instrument Co. Ltd., Dandong, China) to confirm the presence of phases and their crystallinity. Nitrogen adsorption/desorption isotherm curves were obtained using a Micromeritics ASAP-2020M nitrogen adsorption/desorption apparatus (Best Instrument Technology Co. Ltd., Beijing, China) to evaluate porosity. The microstructures and morphologies of the materials were observed using a JSM-6700F scanning electron microscope (SEM) (JEOL, San Hawk, Tokyo, Japan) equipped with an IE300X energy-dispersive X-ray spectrometer and a JEM-2100F transmission electron microscope (TEM) (JEOL, San Hawk, Tokyo, Japan). X-ray photoelectron spectroscopy (XPS) curves were obtained by applying an electron spectrometer (ESCALab250) (Thermo Fisher Scientific, Boston, MA, USA) to analyze the surface of the composites. Raman spectra were collected by using a JobinYvon HR800 Raman spectrometer (Renishaw, Gloucestershire, London, UK).

### 2.4. Electrochemical Measurements

CR2016 coin-type cells were assembled in a glove box under an inert atmosphere without water and oxygen to test the electrode performance. Polypropylene films were used as separators (the thickness of the separator was 25 µm) between the working and counter electrode (lithium wafer) in the electrolyte. An amount of 1 M LiPF_6_ dissolved in the solvent of ethylene carbonate (EC) and dimethyl carbonate (DMC) was used as the electrolyte (1:1, *v*:*v*). The electrode was made by applying a coating slurry of the above active materials, conductive carbon black, and PVDF binder (7:2:1, *w*:*w*:*w*) to the copper foil (the mass loading of the electrode was about 1.2 mg·cm^−2^), which was then dried under vacuum conditions at 120 °C for 14 h. Finally, the copper foil was cut into wafers with uniform size of 9 μm. Galvanostatic cycling measurements were conducted using a CT2001A battery tester (Wuhan LAND Electronic Co. Ltd., Wuhan, China) at specific voltage windows. Electrochemical impedance spectroscopy characteristics and cyclic voltammogram (CV) were obtained by using an electrochemical workstation (Shanghai Chenhua Instrument Co. Ltd., Shanghai, China) with a fixed voltage range and scan rate.

## 3. Results and Discussions

**Figure 1:**
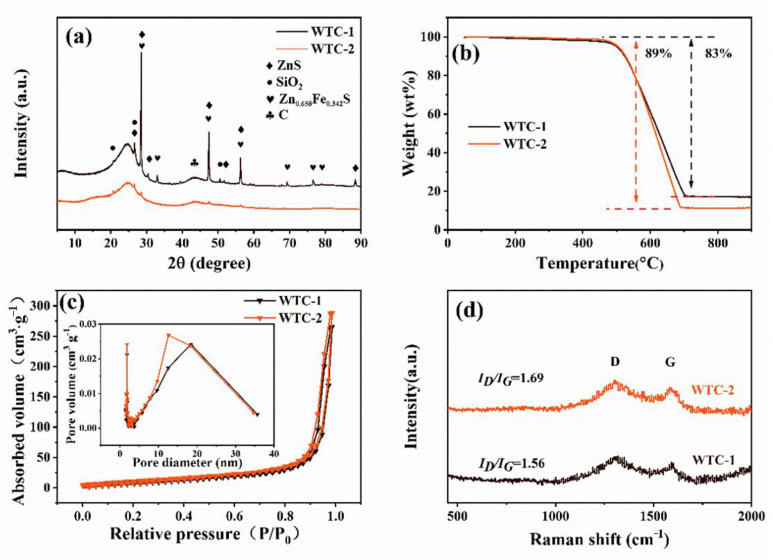
XRD patterns (**a**); TGA curves (**b**); N_2_ adsorption-desorption plots; (**c**) and Raman spectra (**d**) of the prepared materials WTC-1 and WTC-2.

XRD measurements were performed to determine composition. Figure S1 shows the XRD pattern of the waste tire rubber raw powders, in which we clearly observed the diffraction peaks of silicon dioxide (SiO_2_), zinc oxide (ZnO), calcium carbonate (CaCO_3_), sulfur, and iron oxide [19,20]. Additionally, a wide diffraction peak positioned around 25° demonstrates the presence of amorphous carbon. Figure 1a shows the XRD patterns of the processed products. The XRD pattern of WTC-1 XRD indicates the presence of SiO_2_, ZnS, and CaO [19,21,22]. Peaks at 2θ = 26.9, 30.5, 47.5, and 51.7° are attributed to reflections from the (100), (101), (110), and (103) planes of ZnS (JCPDS No. 79-2204), respectively, and ZnS was generated via the chemical reaction between H_2_S and ZnO [23]. For WTC-1, the WTC-2 was further washed by an acid solution, thus the peak of ZnS exhibits a relatively weaker intensity, but small amount of ZnS still exists. Thermogravimetric analysis (TGA) was used to confirm the proportion of carbon and other materials (Figure 1b). Carbon combustion began at around 500 °C and ended at 800 °C. The carbon contents of WTC-1 and WTC-2 were determined to be 83 and 89%, respectively. This demonstrated that the final hydrochloric acid treatment was effective to remove impurities in the resulting carbon materials.

The Brunauer-Emmett-Teller (BET) specific surface area and pore volume for both samples are illustrated in Table S1. Compared with WTC-1, WTC-2 has a relatively higher specific surface area of 46.01 m^2^·g^−1^ and a pore volume of 0.46 cm^3^·g^−1^, which is ascribed to the mesopores and the increase of surface area during the final acid washing step. The nitrogen adsorption–desorption isotherm curves of both samples are shown in Figure 1c. All curves exhibit a distinct hysteresis loop, suggesting the existence of a mesoporous structure. The mesoporous structure in rubber powder carbon is caused by SO_2_ and steam produced during carbonization [24]. In Figure 1d, two broad bands around 1340 and 1590 cm^−1^ were observed in both WTC-1 and WTC-2, representing the D and G band [25], respectively, and the intensity ratio of the D band to the G band (*I_D_*/*I_G_*) represents the degree of graphitization. The calculated ratio, *I_D_*/*I_G_* is approximately 1.56 and 1.69 for WTC-1 and WTC-2, respectively, further demonstrating the partial graphitization of the waste tire carbon. The increase of the *I*_D_/*I*_G_ is due to the cleaning of impurities, which leads to the defects of carbon structure. 

**Figure 2:**
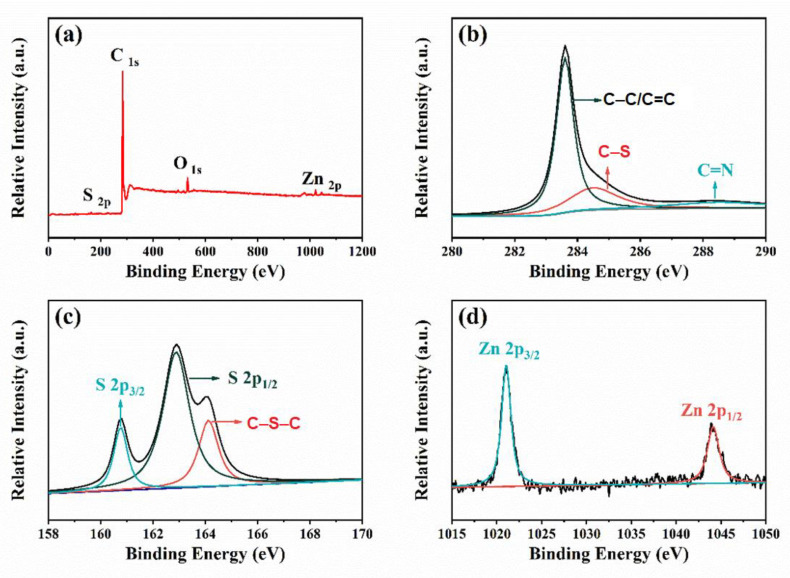
XPS spectra of (**a**) WTC-2; (**b**) C 1s; (**c**) S 2p and (**d**) Zn 2p.

The chemical states of elements for WTC-2 powders were further investigated by XPS, which are shown in Figure 2. The full XPS spectra denote the presence of elements including Zn, O, C, and S, consistent with XRD results. The O 1s spectrum of all harvested materials is shown in Figure S2. As indicated in Figure 2b, three peaks at 531.9, 533.4, and 536.0 eV were attributed to C=O, C–OH, and O=C–OH [26,27]. The C 1s spectrum is divided into three peaks with binding energies of 286.3, 285.4, and 284.6 eV, corresponding to C–S, C=N, and C–C/C=C [28]. In the S 2p spectrum (Figure 2c), the peaks at 160.5 and 162.7 eV correspond to S 2p_1/2_ and S 2p_3/2_, respectively, whereas the peak at 164.08 eV is attributed to C–S–C bonding [29,30,31,32]. In Figure 2d, the presence of bivalent Zn is confirmed: the peaks at around 1045 and 1023 eV are assigned to the Zn 2p_1/2_ and Zn 2p_3/2_ components, respectively [33,34].

**Figure 3:**
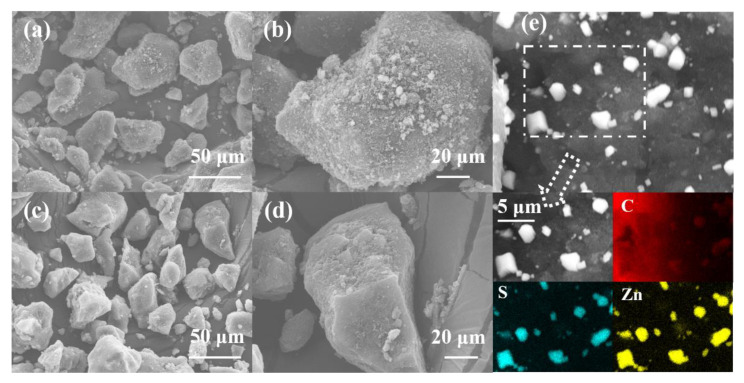
SEM micrographs of (**a**,**b**) WTC-1; (**c**,**d**) WTC-2 and (**e**) EDS elemental mapping of WTC-1.

Figure 3 shows SEM micrographs of the recovered carbon materials. For WTC-1, the bulk materials exhibited a rough surface covered with small particles (Figure 3a,b). Compared with WTC-1, there are fewer small particles on the surface for WTC-2, confirming that the final acid washing procedure has removed most impurities (Figure 3c,d). Figure S5 shows the size distribution of samples WTC-1 and WTC-2. The samples obtained before and after the acid washing procedure have similar size. The EDS mapping of WTC-1 is shown in Figure 3e, demonstrating that the main components of WTC-1 are C, O, Zn, and S, as well as other elements such as Fe, Ca, and Si (shown in Figure S3), which demonstrates the coexistence and homogeneous distribution of C, Zn, and S throughout the sample.

**Figure 4:**
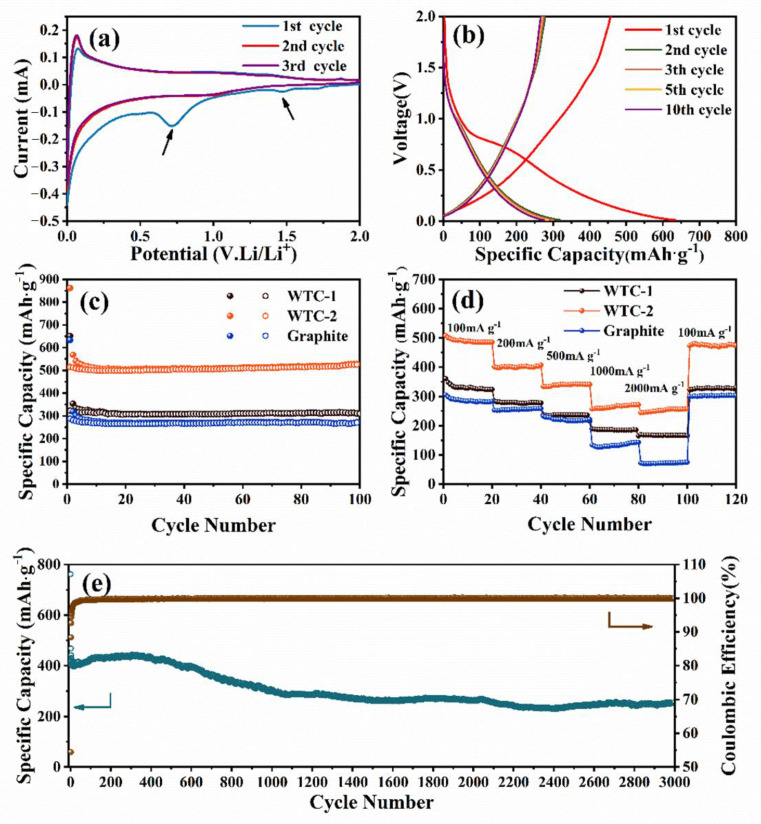
Electrochemical properties of the prepared samples: cyclic voltammograms for the first three cycles of (**a**) WTC-2 at a scan rate of 0.1 mV·s^−1^ for the range 0–2.0 V; (**b**) initial discharge-charge curves at 100 mA·g^−1^; (**c**) cycling performance at 100 mA·g^−1^; (**d**) rate performances of WTC-1, WTC-2, and graphite and (**e**) long-term cyclic performance of WTC-2 at 1000 mA·g^−1^.

Figure 4a shows the cyclic voltammetry (CV) curve of WTC-2 for the first three cycles. In the first cathodic scan, two distinct peaks observed at 0.72 and 1.45 V are attributed to the production of a solid electrolyte interface (SEI) film [35,36,37] and the generation of a Li-Zn alloy [38,39]. In addition, in the following anodic scan, the peak at 1.3 V is related to the oxygenation of metallic Zn to ZnS. In the whole process, there is the equation: (1) 2Li + ZnS → Li_2_S + Zn (1.45 V); (2) Li_2_S + Zn → ZnS + 2Li (1.3 V) [40]. The cathodic peak at 0.1 V and anodic peak at 0.2 V can be attributed to the processes of Li ions insertion and extraction of carbon. As the process of SEI film formation occurs during the first cycle, the 0.72 V peak disappeared during the second cycle. No distinct variations in CV curves were observed during the following scans, confirming the high reversibility and outstanding cycling stability of the waste tire carbon.

The galvanostatic charge/discharge curves of WTC-2 over ten cycles are shown in Figure 4b. The initial specific discharge and charge capacities of the WTC-2 electrode were 633.5 and 457.2 mAh·g^−1^, respectively, presenting the initial coulombic efficiency of 72.1%. Capacity loss during the first cycle occurred due to the formation of an SEI film and the irreversible reaction of impurities during discharge. After the first cycle, the coulombic efficiency increased to 87.4% in the second cycling and reached 96.7% after the tenth cycle. The plateau at 0.7 V was not observed in subsequent cycles, consistent with the aforementioned CV results.

Figure 4c shows the cycling behavior of WTC-1, WTC-2, and graphite at 100 mA·g^−1^. Compared with commercial graphite, WTC-2 exhibits obviously higher specific capacities and better cyclic behavior, in particular, WTC-2 maintains a charge capacity of 525.6 mAh·g^−1^ after 100 cycles. Studies have shown that the presence of small amounts of impurity ZnS as active substance conducive to increase the battery’s capacity [40]. Contrastive experiments further prove this conclusion. Figure S4 shows the ZnS/C and commercial graphite at 100 mA·g^−1^, the charge capacity of which after 100 cycles is enhanced to 353.7 mAh·g^−1^. Similarly, ZnS as an active material in ZnS/C is also conducive to improve the capacity, the principle is decomposition of ZnS into Zn and the formation of Li_2_S [15]. The rate performances of WTC-1, WTC-2, and commercial graphite were evaluated by cycling the electrodes at different current densities (Figure 4d), which demonstrated that WTC-2 presents superior rate capability, with corresponding reversible capacities of 485.7, 403, 340.9, 271.6, and 257.4 mAh·g^−1^ at currents of 100, 200, 500, 1000, and 2000 mA·g^−1^, respectively. Even at a high current of 2000 mA·g^−1^, the reversible specific capacity stabilized at 271.6 mAh·g^−1^ after twenty cycles. Upon recovering to 100 mA·g^−1^, the WTC-2 electrode returned a capacity of 474.6 mAh·g^−1^, indicating the high cycling stability and good rate capability. The long-term cycling behavior of WTC-2 at 1000 mA·g^−1^ is indicated in Figure 4e, showing that its reversible specific capacity is maintained at 255.1 mAh·g^−1^ after 3000 cycles, and demonstrating an excellent level stability for use as an anode in LIBs. Figure S6 shows the SEM image of WTC-2 after 3000 cycles at 1000 mA·g^−1^. No cracking and exfoliation were found in the samples, which also proved the structural stability.

**Figure 5:**
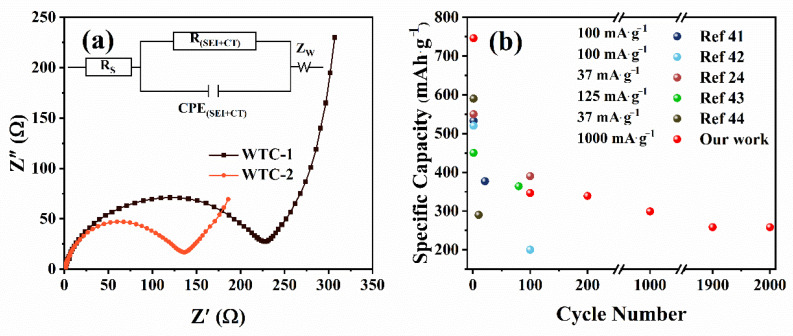
(**a**) Electrochemical impedance plots of WTC-1 and WTC-2; insert shows the equivalent circuit of the Nyquist plot of WTC−2 and (**b**) compares its electrochemical performance with previous reports of waste tire carbon.

Figure 5a shows the Nyquist plots of WTC-1 and WTC-2. Each impedance spectra consists of two main zones: a semicircle in the high-frequency region, which is associated with charge transfer resistance, and a sloping straight line in the lower frequency region, representing Li^+^ diffusion in the bulk of the electrode. WTC-2 is characterized by a Nyquist plot with a smaller semicircle, denoting lower charge transfer resistance and higher electronic conductivity. The equivalent circuit of the Nyquist plot of WTC-2 is provided in Figure 5a to imitate the impedance plots. Here, R_S_, R_SEI_, and R_CT_ represent electrolyte resistance (Li^+^ transfer through the electrolyte to the electrode surface), surface resistance (Li^+^ entering the SEI layer), and charge transfer resistance, respectively. Z_W_ represents the Warburg impedance, which correlates to the total Li^+^ diffusing into the interspace of the active materials. Finally, we compared WTC-2 against numerous other reported carbon materials derived from waste tires as anodes for LIBs. Figure 5b shows the comparison of WTC-2 with other waste tire carbon recently reported. [24,41,42,43,44]. It can be indicated that the specific capacities of literature [41] and [42] are 540 and 530 mAh·g^−1^, respectively, at 100 mA·g^−1^. However, the specific capacity of WTC-2 is 750 mAh·g^−1^ at 1000 mA·g^−1^. Moreover, there is no relevant research for cycling stability over 100 cycles reported. The specific capacity of WTC-2 remains at 255.1 mAh·g^−1^ after 2000 cycles, indicating an excellent level of stability (Figure 4e). Apparently, WTC-2 presents a strong advantage of cycling stability and superior specific capacity.

## 4. Conclusions

In summary, we demonstrated a facile route for the recovery of high-performance carbon from waste tire rubber as a potential anode for LIBs. The composite delivers a specific capacity as high as 255.1 mAh·g^−1^ after 3000 cycles at a current density of 1000 mA·g^−1^. Although residual ZnO in rubber powder generates ZnS in the simple process of carbonization, its existence has a positive effect on the electrochemical performance. The finding that waste tire can be successfully used to manufacture anodes of LIBs has a very meaningful impact on the recycling use of waste tires and reduces the environmental impact of these materials.

## Data Availability

The data presented in this study are available on request from the corresponding author after obtaining permission from an authorized person.

## Supplementary Materials

The following are available online at https://www.mdpi.com/article/10.3390/ma14092178/s1, Figure S1: XRD patterns of waste tire rubber powders. Figure S2: XPS spectrum of O 1s of WTC-2. Figure S3: Corresponding element mapping of Fe, Si, O, and Ca in WTC-1. Figure S4: The cycle performance of ZnS/C and commercial graphite at a current density of 100 mA g−1. Figure S5: Size distribution of samples WTC-1 and WTC-2. Figure S6: (**a**) The picture and (**b**) SEM image of WTC-2 after 3000 cycles at 1000 mA·g−1. Table S1: The Brunauer-Emmett-Teller (BET) surface area, pore volume and average pore size of the samples.
