# Supplementary material for: The Positive Effect of ZnS in Waste Tire Carbon as Anode for Lithium-Ion Batteries
Source: Materials (Basel). 2021 Apr 24;14(9):2178. doi: 10.3390/ma14092178 (PMC8122980; doi:10.3390/ma14092178)
Supplement: Supplementary file 1 [file materials-14-02178-s001.zip › materials-1188152-supplementary.pdf]

materials

Supplementary Material

The Positive Effect of ZnS in Waste Tire Carbon as Anode for Lithium-Ion Batteries

Xuechen Wang [1,†], Lu Zhou [2,†], Jianjiang Li [3], Na Han [1], Xiaohua Li [1], Gang Liu [3], Dongchen Jia [3], Zhaoli Ma [4], Guojun Song [1], Xiaoyi Zhu [3], Zhi Peng [1,*] and Lei Zhang [5,*]

1 School of Material Science and Engineering, Qingdao University, No. 308, Ningxia Road Qingdao 266071, China; 2018020384@qdu.edu.cn (X.W.); 2018020395@qdu.edu.cn (N.H.); 2019020442@qdu.edu.cn (X.L.); songguojunqdu@126.com (G.S.)
2 College of Chemistry and Chemical Engineering, Qingdao University, No. 308, Ningxia Road Qingdao 266071, China; zhoulu137@126.com
3 School of Environmental Science and Engineering, Qingdao University, No. 308, Ningxia Road Qingdao 266071, China; jjli@qdu.edu.cn (J.L.); 2019025785@qdu.edu.cn (G.L.); 2020025847@qdu.edu.cn (D.J.); xyzhu@qdu.edu.cn (X.Z.)
4 Chemical Experimental Teaching Center, Qingdao University, No. 308, Ningxia Road Qingdao 266071, China; zlma@qdu.edu.cn
5 Centre for Clean Environment and Energy, Griffith University, Gold Coast Campus, Gold Coast, QLD 4222, Australia
* Correspondence: pengzhi@qdu.edu.cn (Z.P.); lei.zhang@griffith.edu.au (L.Z.)
† These authors equally contributed to this work

Citation: Wang, X.; Zhou, L.; Li, J.; Han, N.; Li, X.; Liu, G.; Jia, D.; Ma, Z.; Song, G.; Zhu, X.; et al. The positive effect of ZnS in waste tire carbon as anode for lithium-ion batteries. *Materials* **2021**, *14*, 2178. https://doi.org/10.3390/ma14092178

Academic Editor: Jean-Francois Drillet

Received: 1 April 2021
Accepted: 21 April 2021
Published: 24 April 2021

Publisher's Note: MDPI stays neutral with regard to jurisdictional claims in published maps and institutional affiliations.

Copyright: © 2021 by the authors. Submitted for possible open access publication under the terms and conditions of the Creative Commons Attribution (CC BY) license (http://creativecommons.org/licenses/by/4.0/).

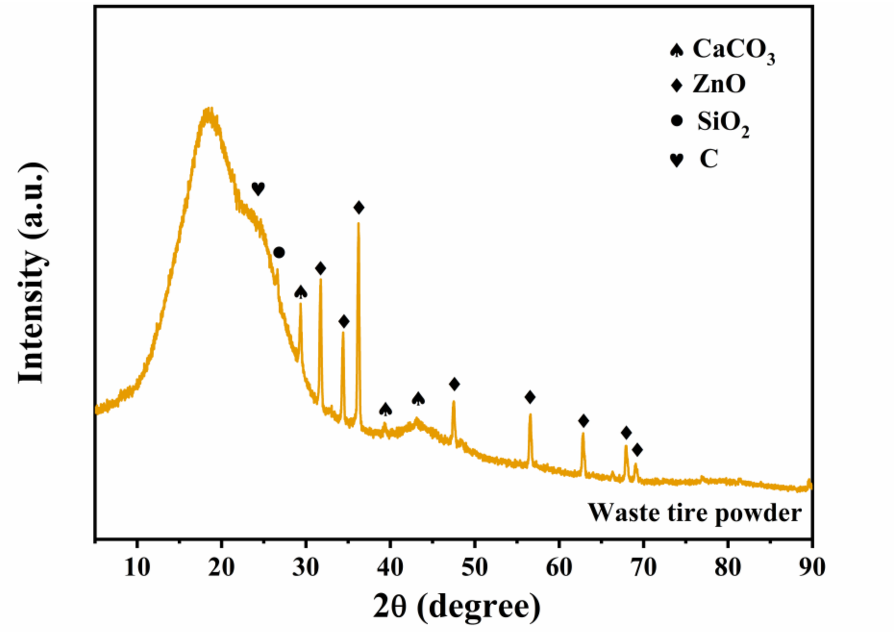

Figure S1. XRD patterns of waste tire rubber powders.

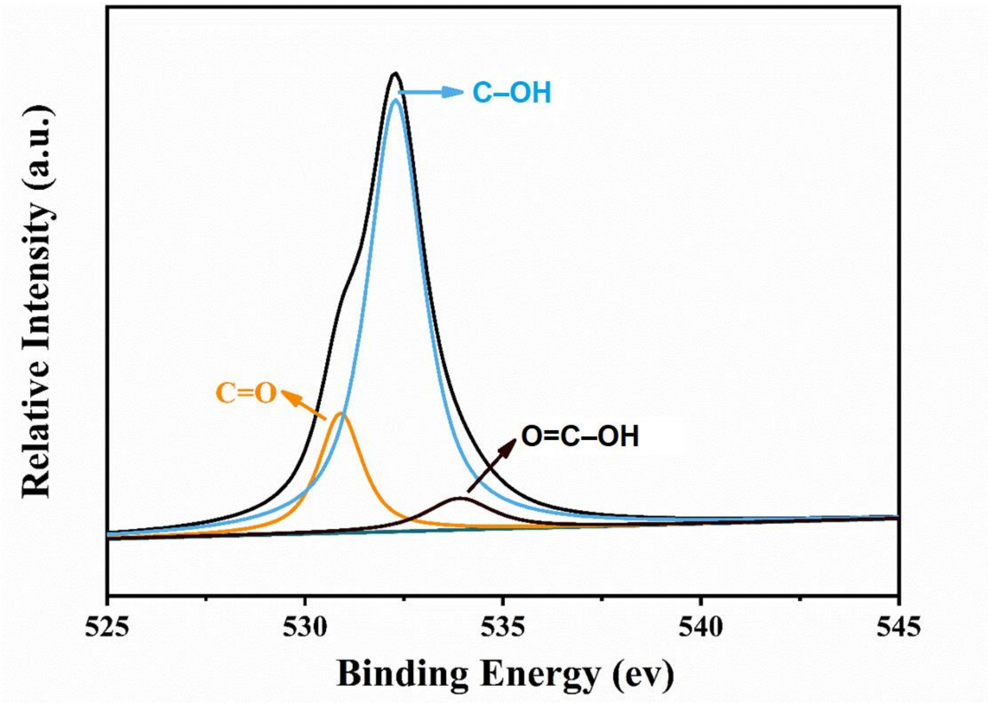

Figure S2. XPS spectrum of O 1s of WTC-2.

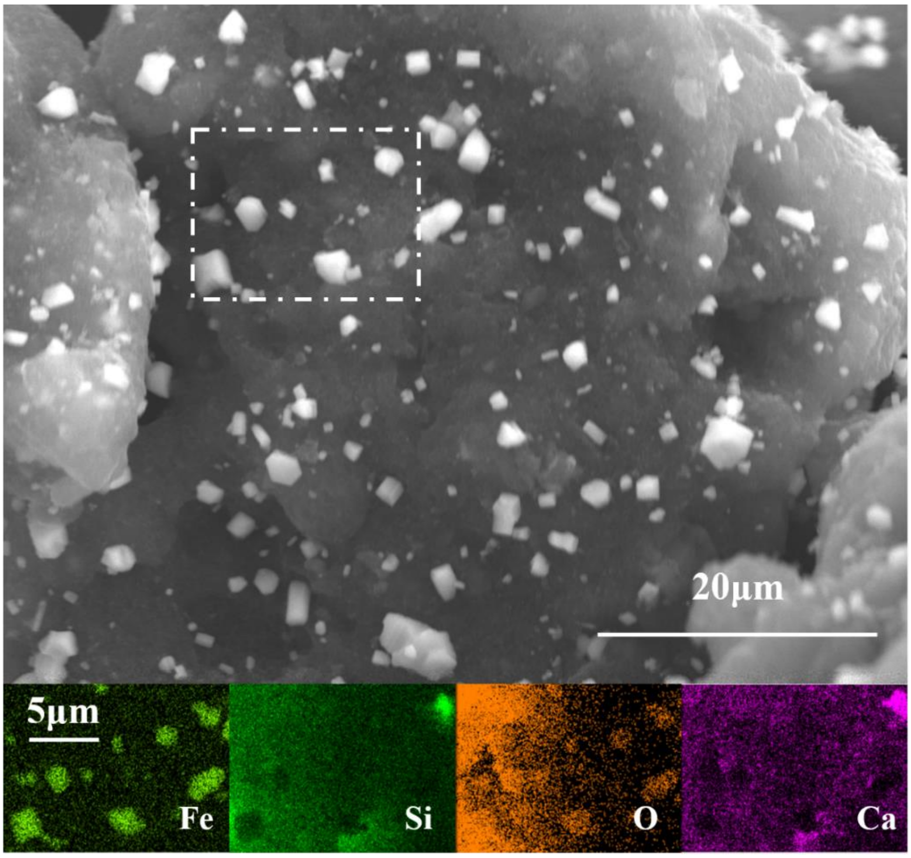

Figure S3. Corresponding element mapping of Fe, Si, O, and Ca in WTC-1.

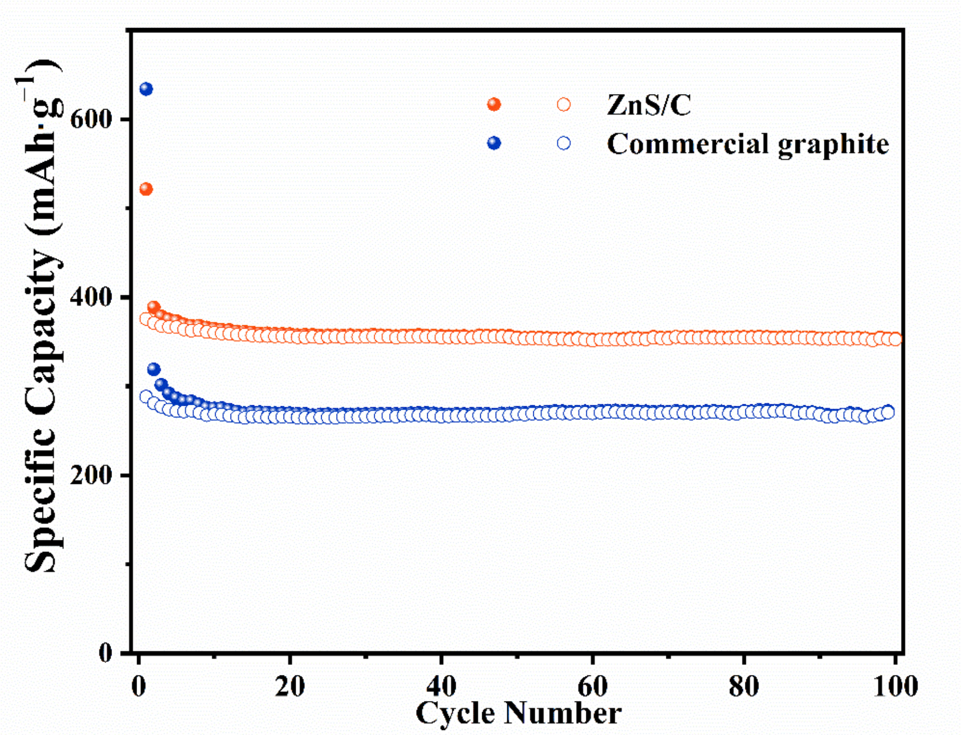

Figure S4. The cycle performance of ZnS/C and commercial graphite at a current density of 100 mA·g^{-1}.

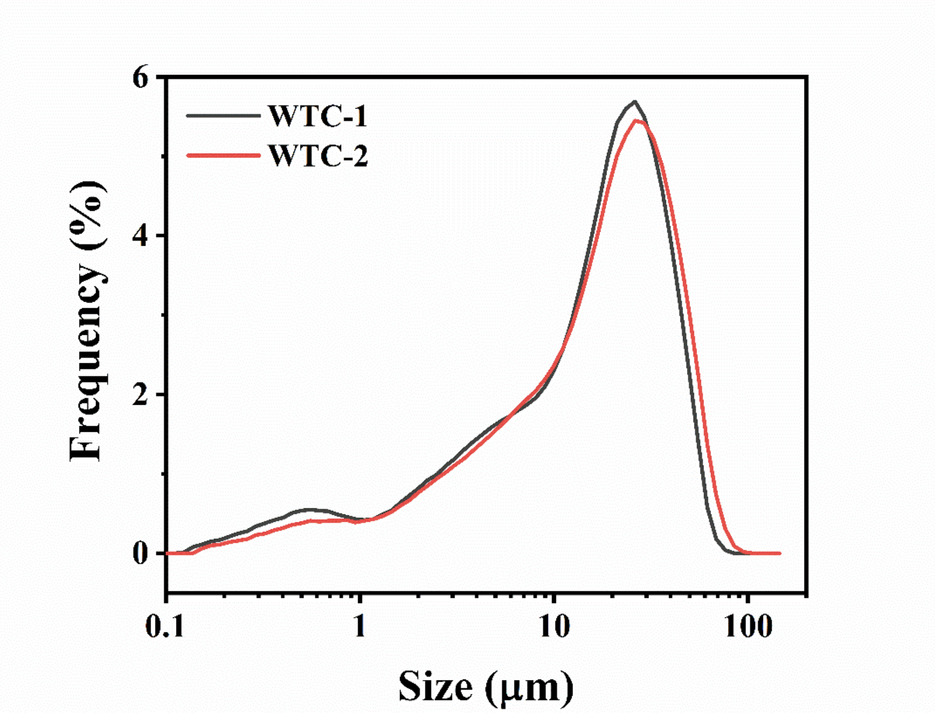

Figure S5. Size distribution of samples WTC-1 and WTC-2.

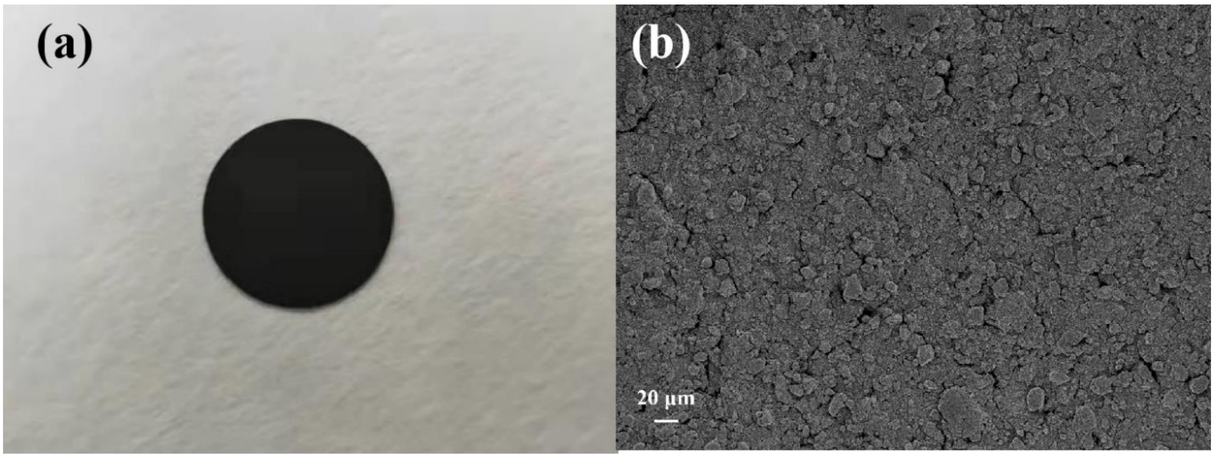

Figure S6. (**a**) The picture and (**b**) SEM image of WTC-2 after 3000 cycles at 1000 mA·g^{-1}.

Table S1. The Brunauer-Emmett-Teller (BET) surface area, pore volume and average pore size of the samples.

**Sample**	**WTC-1**	**WTC-2**
Surface area (m^2·g^{-1})	38.07	46.01
Pore volume (cm^3·g^{-1})	0.41	0.46
Average pore size (nm)	2.16	1.94
